# Supplementary material for: Optimizing hepatitis C virus testing in the era of point-of-care RNA diagnostics
Source: J Clin Microbiol. 2025 Dec 17;64(1):e01259-25. doi: 10.1128/jcm.01259-25 (PMC12802147; doi:10.1128/jcm.01259-25)
Supplement: Figure S1 — HCV RNA tests by viral load based on a cutoff of 10,000 IU/mL. [file jcm.01259-25-s0001.pdf]

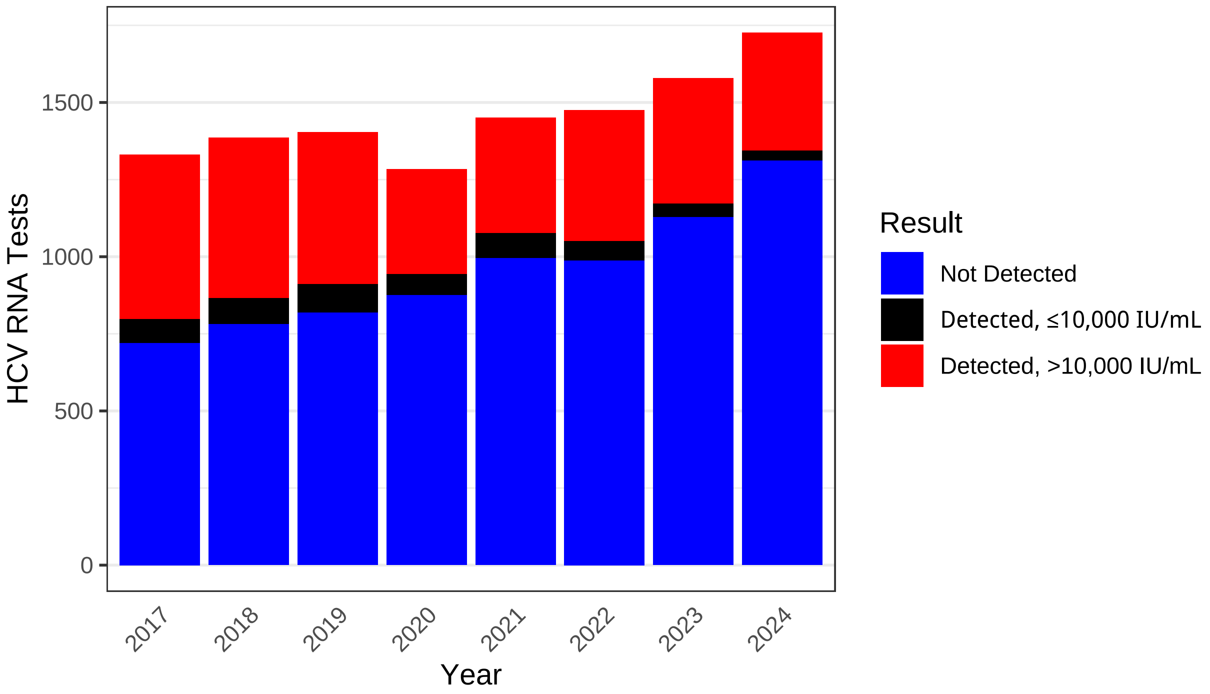

Supplemental Figure 1. The percentage of reflex HCV RNA tests that are detectable but less than 10,000 IU/mL has decreased by 2.8 fold from 2017 to 2024. Results from HCV RNA tests that were ordered as a reflex from a positive antibody screen were grouped according to result.
